# HPLC profiles and spectroscopic data of cassane-type furanoditerpenoids

**DOI:** 10.1016/j.dib.2018.10.068

**Published:** 2018-10-25

**Authors:** Yui Akihara, Sayuri Kamikawa, Yui Harauchi, Emi Ohta, Tatsuo Nehira, Hisashi Ômura, Shinji Ohta

**Affiliations:** a Graduate School of Biosphere Science, Hiroshima University, 1-7-1 Kagamiyama, Higashi-Hiroshima 739-8521, Japan; b Graduate School of Integrated Arts and Sciences, Hiroshima University, 1-7-1 Kagamiyama, Higashi-Hiroshima 739-8521, Japan

**Keywords:** Sulcobruchus sauteri, Caesalpinia decapetala, Cassane diterpenoid, HPLC, NMR, ESIMS

## Abstract

The data presented here are related to the research paper entitled “Hydroxylated furanoditerpenoids from the pupal case produced by the bruchid beetle *Sulcobruchus sauteri* inside the seed of *Caesalpinia decapetala*” (Akihara et al., 2018) [1]. In this data article, we provide high-performance liquid chromatography (HPLC) profiles of seven undescribed hydroxylated furanoditerpenoids, caesalsauteolide, 2-hydroxycaesaljapin, 2,7-dihydroxycaesaljapin, 2-hydroxycaesalacetal, caesalsauterol, 6-acetylcaesalsauterol, norcaesalsauterol isolated from the pupal cases produced by *Sulcobruchus sauteri* and four known compounds, caesaljaponin A (Kamikawa et al., 2015) [2], caesaljaponin B (Kamikawa et al., 2015) [2], caesalacetal (Kamikawa et al., 2016) [3], and caesaljapin (Kamikawa et al., 2016; Ogawa et al., 1992) [3], [4] isolated from the cotyledons of the intact seeds of *Caesalpinia decapetala*. Besides, 1D NMR, 2D NMR, and HRESIFTMS data of the seven undescribed furanoditerpenoids are also presented.

**Specifications table**

**Table:** 

Subject area	*Chemistry*
More specific subject area	*Natural products*
Type of data	*Figure*
How data was acquired	*NMR spectroscopy: JEOL A400; HRESIMS: Thermo Fisher Scientific LTQ Orbitrap XL mass spectrometer; High-performance liquid chromatography (HPLC)-photodiode array (PDA) analyses: Inertsil ODS-3 column (150*×*4.6 mm i.d., 5* *μm) on a JASCO LC-2000 instrument equipped with a JASCO MD-2015 multiwavelength detector.*
Data format	*Analyzed*
Experimental factors	*The undescribed hydroxylated furanoditerpenoids were purified by column chromatography.*
Experimental features	*The isolated compounds were characterized by HRESIMS and NMR spectroscopy*
Data source location	*Higashi-Hiroshima, Japan*
Data accessibility	*Data are available with this article*
Related research article	*Y. Akihara, S. Kamikawa, Y. Harauchi, E. Ohta, T. Nehira, H. Ômura, S. Ohta, Hydroxylated furanoditerpenoids from pupal cases produced by the bruchid beetle Sulcobruchus sauteri inside the seeds of Caesalpinia decapetala, Phytochemistry 156 (2018) 151–158.*

**Value of the data**

• The data presents HPLC profiles, NMR data, and HRESIMS data of newly isolated furanoditerpenoids and could be used by other researchers.
• The provided information on the spectroscopic data of furanoditerpenoids could be useful for the analysis of spectra and determination of the structure of other furanoditerpenoids.
• This data can serve as a benchmark for other researchers to elucidate the structures of furanoditerpenoids.

## 1 Data

**Fig. 1:**
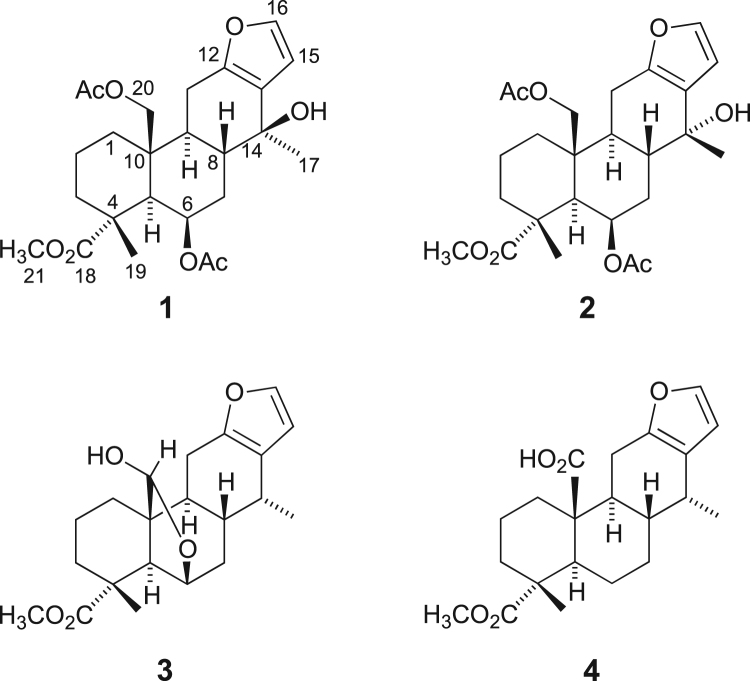
Structures of furanoditerpenoids isolated from the intact seeds of *C. decapetala*.

**Fig. 2:**
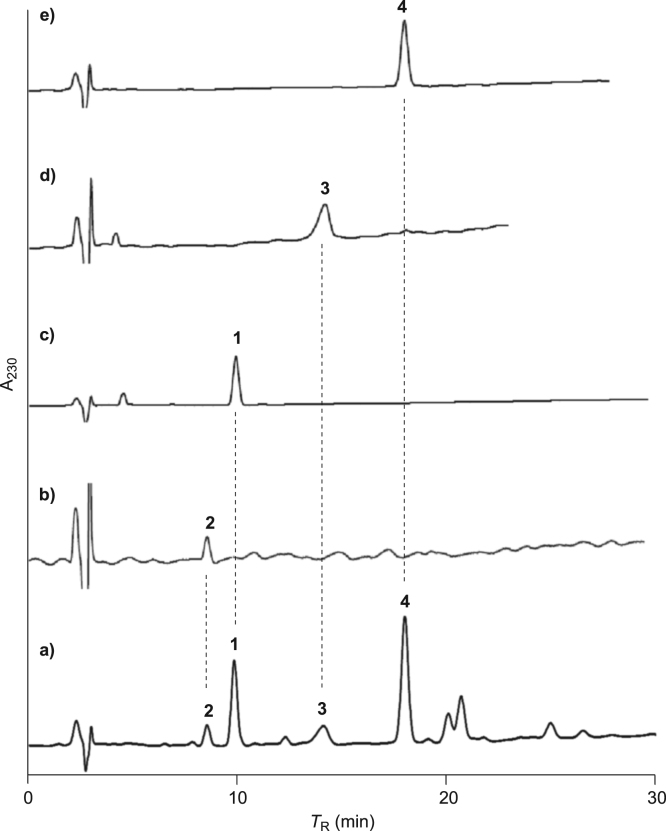
HPLC profiles of a) the EtOAc-soluble fraction of the intact seeds of *C. decapetala*, b) caesaljaponin B (**2**), c) caesaljaponin A (**1**), d) caesalacetal (**3**), and e) caesaljapin (**4**).

**Fig. 3:**
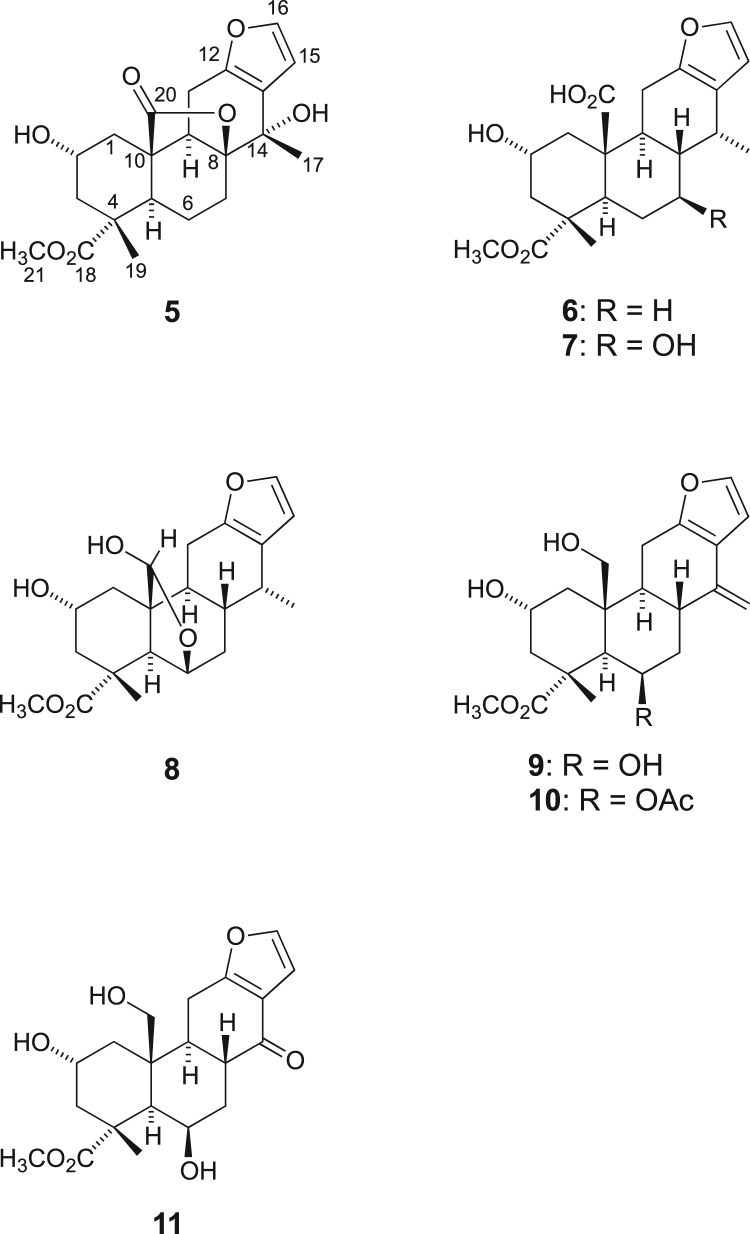
Structures of furanoditerpenoids isolated from the pupal cases produced by *S. sauteri*.

**Fig. 4:**
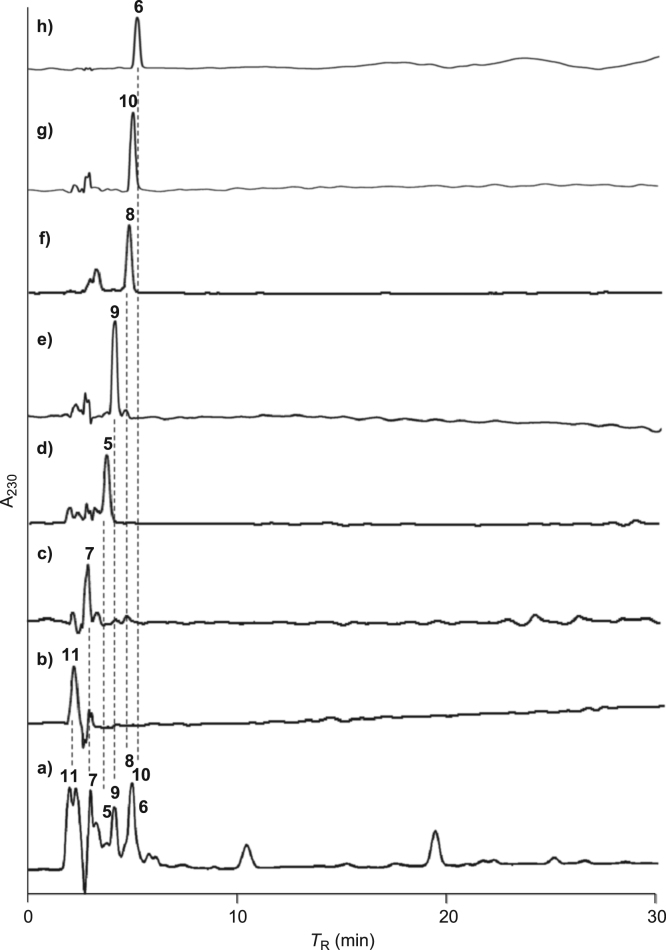
HPLC profiles of a) the EtOAc-soluble fraction of the pupal case produced by *S. sauteri*, b) norcaesalsauterol (**11**), c) 2,7-dihydroxycaesaljapin (**7**), d) caesalsauteolide (**5**), e) caesalsauterol (**9**), f) 2-hydroxycaesalacetal (**8**), g) 6-acetylcaesalsauterol (**10**), and h) 2-hydroxycaesaljapin (**6**).

**Fig. 5:**
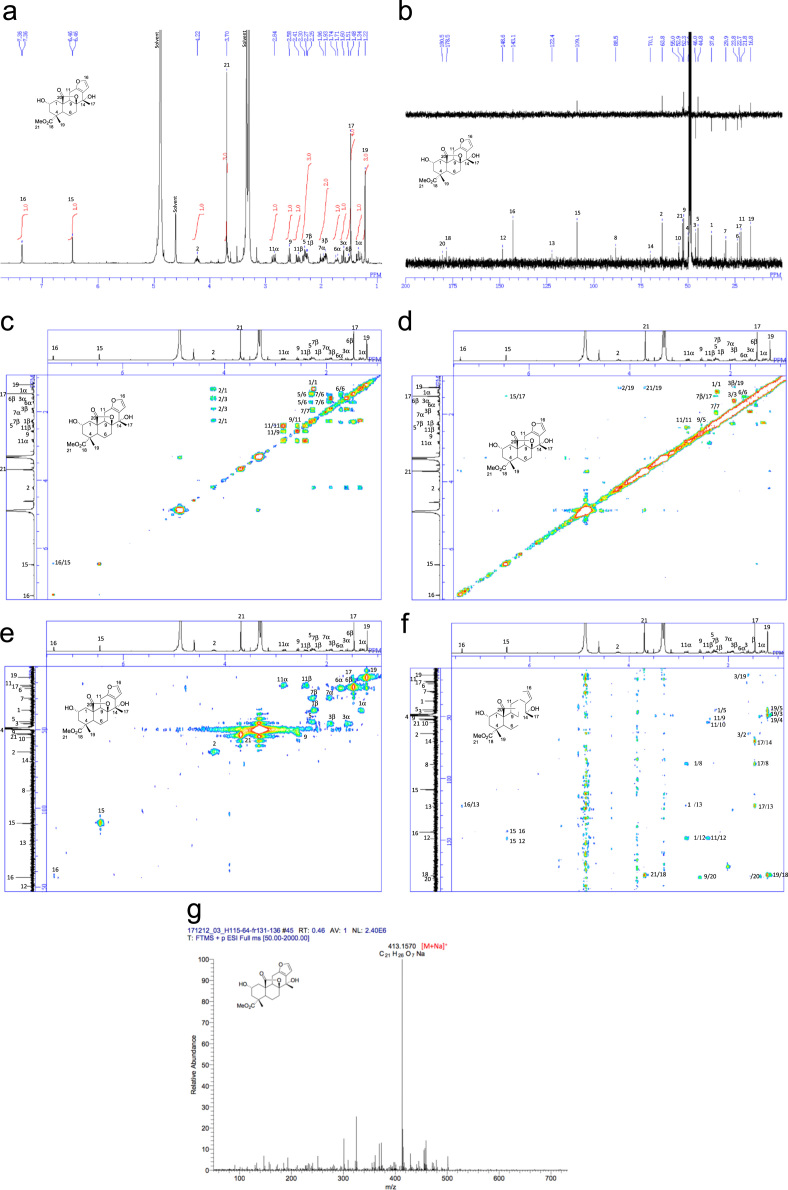
**a.**^1^H NMR (400 MHz, CD_3_OD) of **5. b.**^13^C NMR and DEPT (100 MHz, CD_3_OD) of **5. c.**^1^H-^1^H COSY of **5. d.** NOESY of **5. e.** HSQC of **5. f.** HMBC of **5. g.** (+)HRESIFTMS of **5**.

**Fig. 6:**
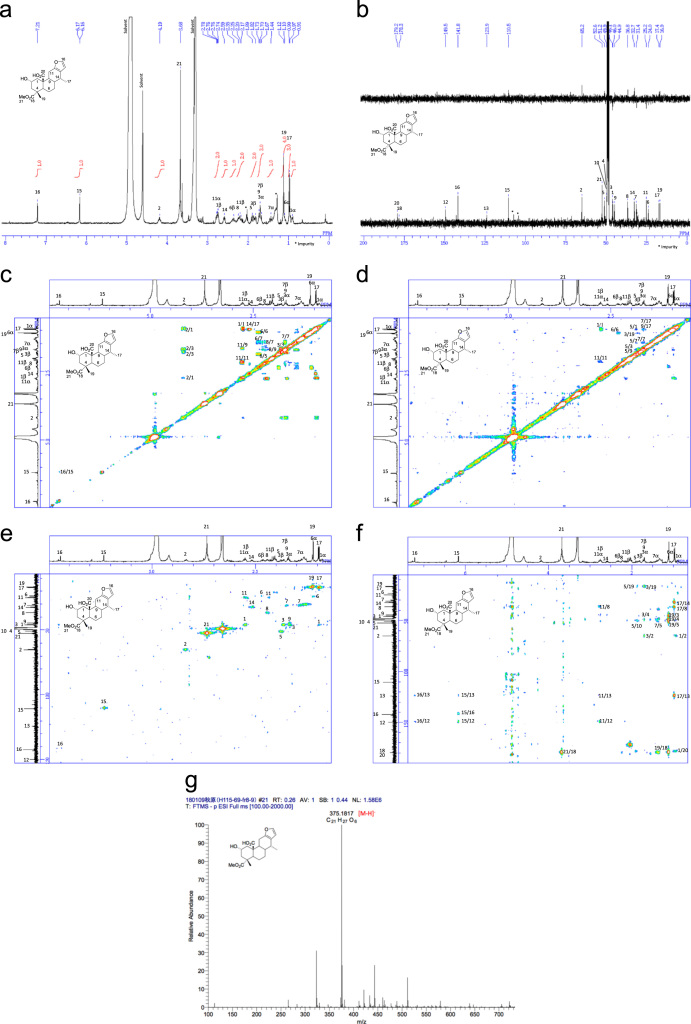
**a.**^1^H NMR (400 MHz, CD_3_OD) of **6. b.**^13^C NMR and DEPT (100 MHz, CD_3_OD) of **6. c.**^1^H-^1^H COSY of **6. d.** NOESY of **6. e.** HSQC of **6. f.** HMBC of **6. g.** (-)HRESIFTMS of **6**.

**Fig. 7:**
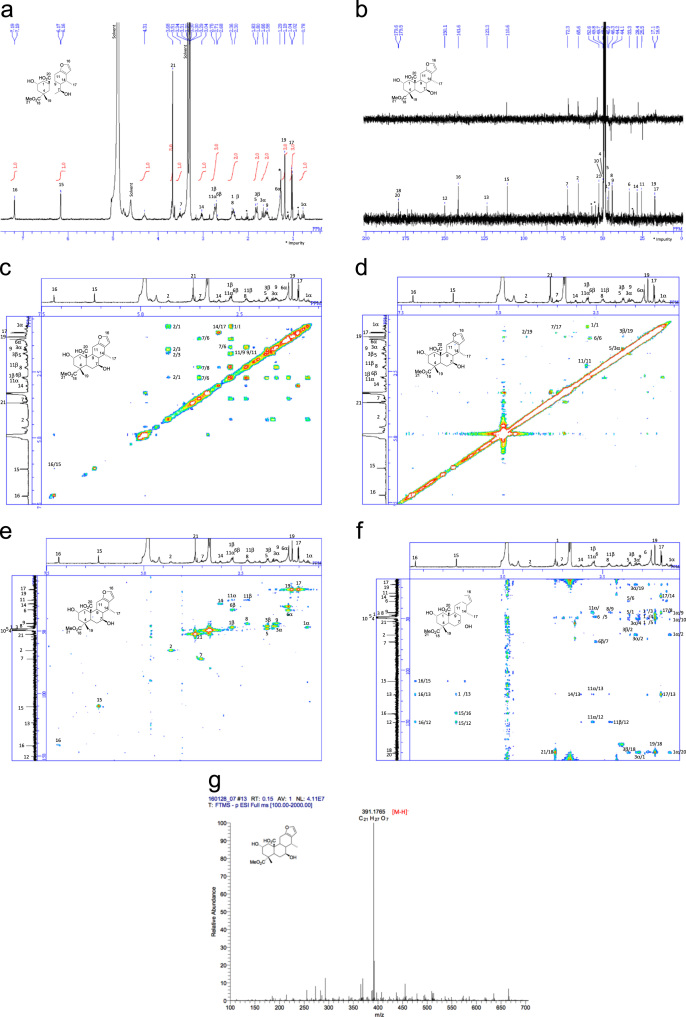
**a.**^1^H NMR (400 MHz, CD_3_OD) of **7. b.**^13^C NMR and DEPT (100 MHz, CD_3_OD) of **7. c.**^1^H-^1^H COSY of **7. d.** NOESY of **7. e.** HSQC of **7. f.** HMBC of **7. g.** (-)HRESIFTMS of **7**.

**Fig. 8:**
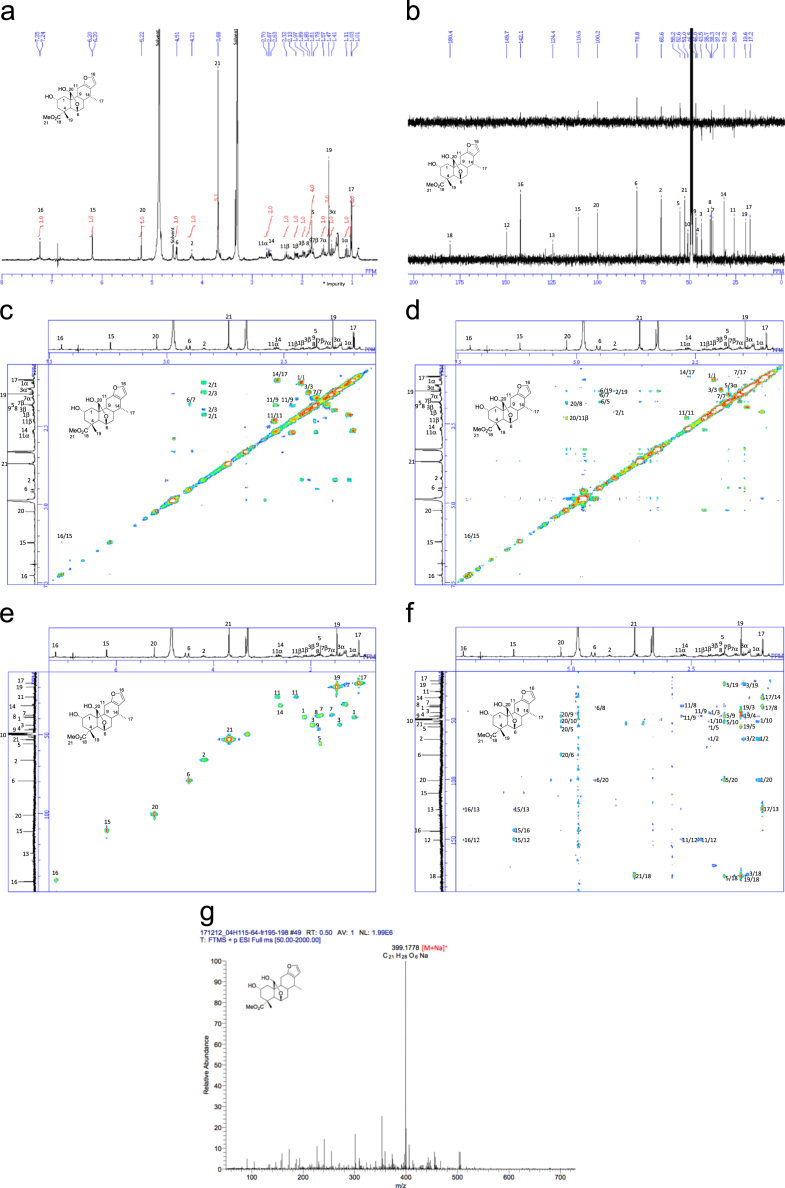
**a.**^1^H NMR (400 MHz, CD_3_OD) of **8. b.**^13^C NMR and DEPT (100 MHz, CD_3_OD) of **8. c.**^1^H-^1^H COSY of **8. d.** NOESY of **8. e.** HSQC of **8. f.** HMBC of **8. g.** (+)HRESIFTMS of **8**.

**Fig. 9:**
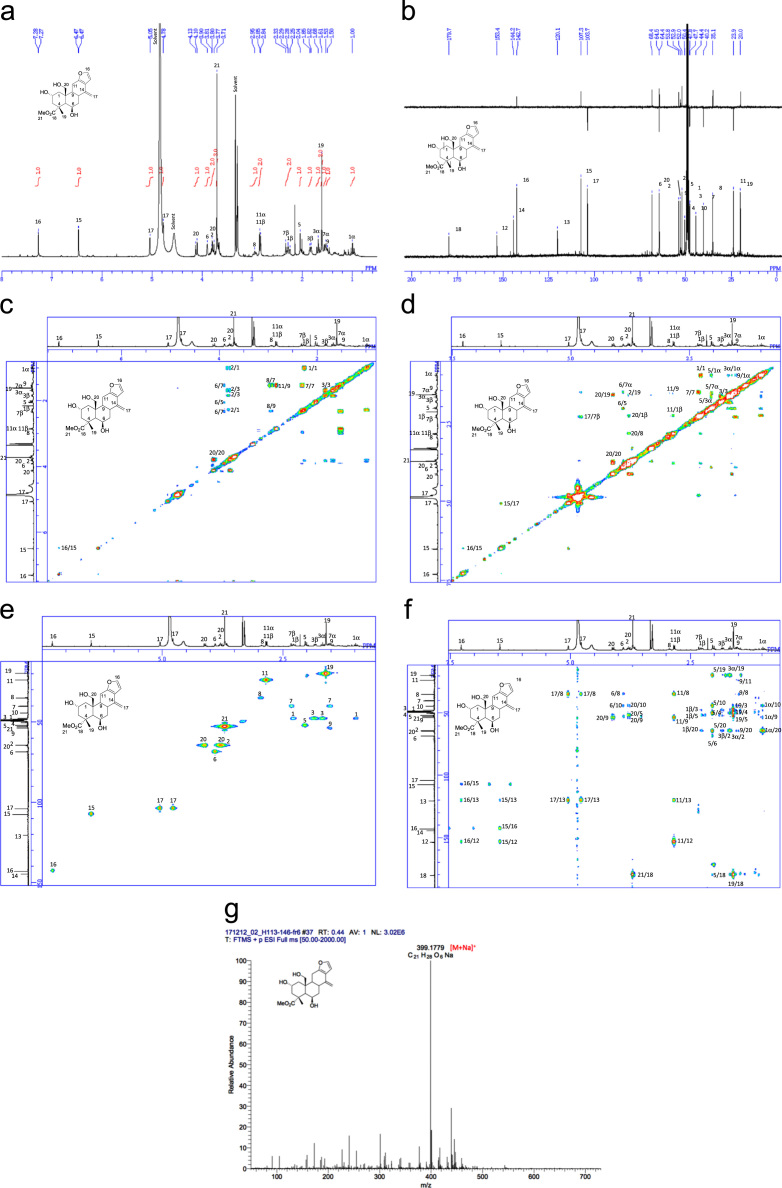
**a.**^1^H NMR (400 MHz, CD_3_OD) of **9. b.**^13^C NMR and DEPT (100 MHz, CD_3_OD) of **9. c.**^1^H-^1^H COSY of **9. d.** NOESY of **9. e.** HSQC of **9. f.** HMBC of **9. g.** (+)HRESIFTMS of **9**.

**Fig. 10:**
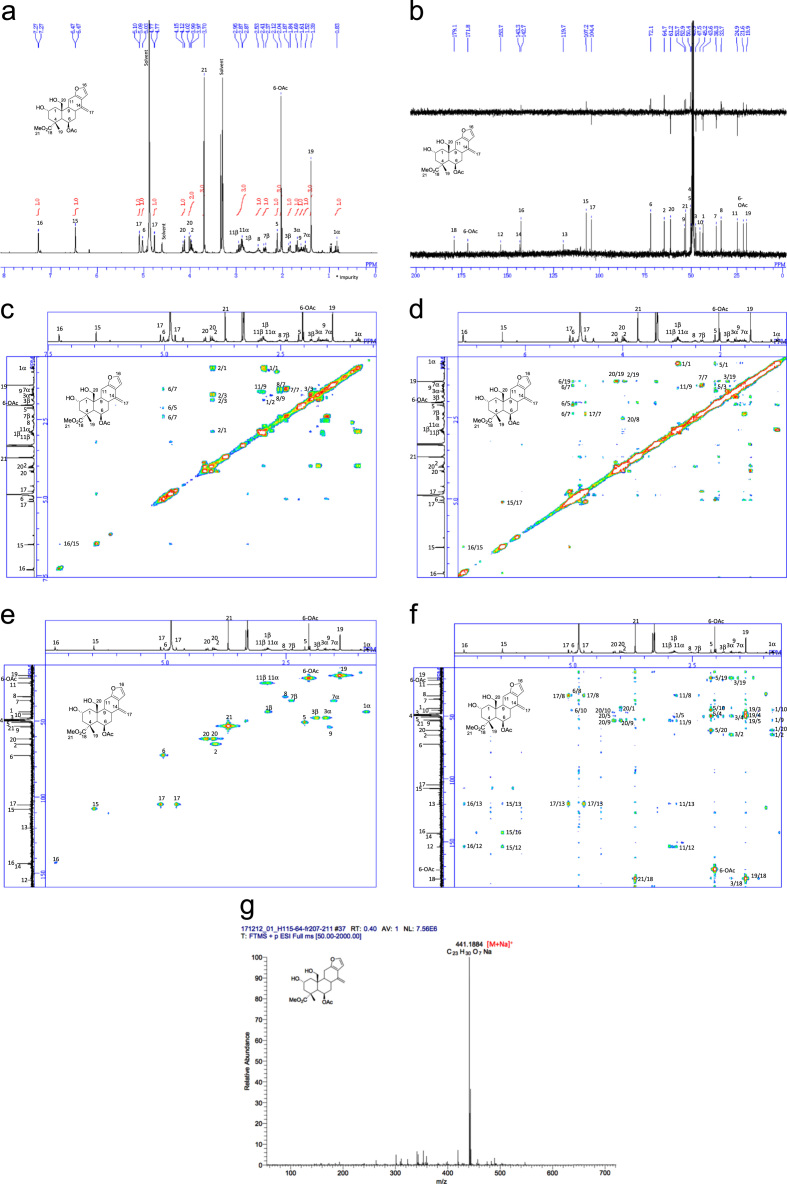
**a.**^1^H NMR (400 MHz, CD_3_OD) of **10. b.**^13^C NMR and DEPT (100 MHz, CD_3_OD) of **10. c.**^1^H-^1^H COSY of **10. d.** NOESY of **10. e.** HSQC of **10. f.** HMBC of **10. g.** (+)HRESIFTMS of **10**.

**Fig. 11:**
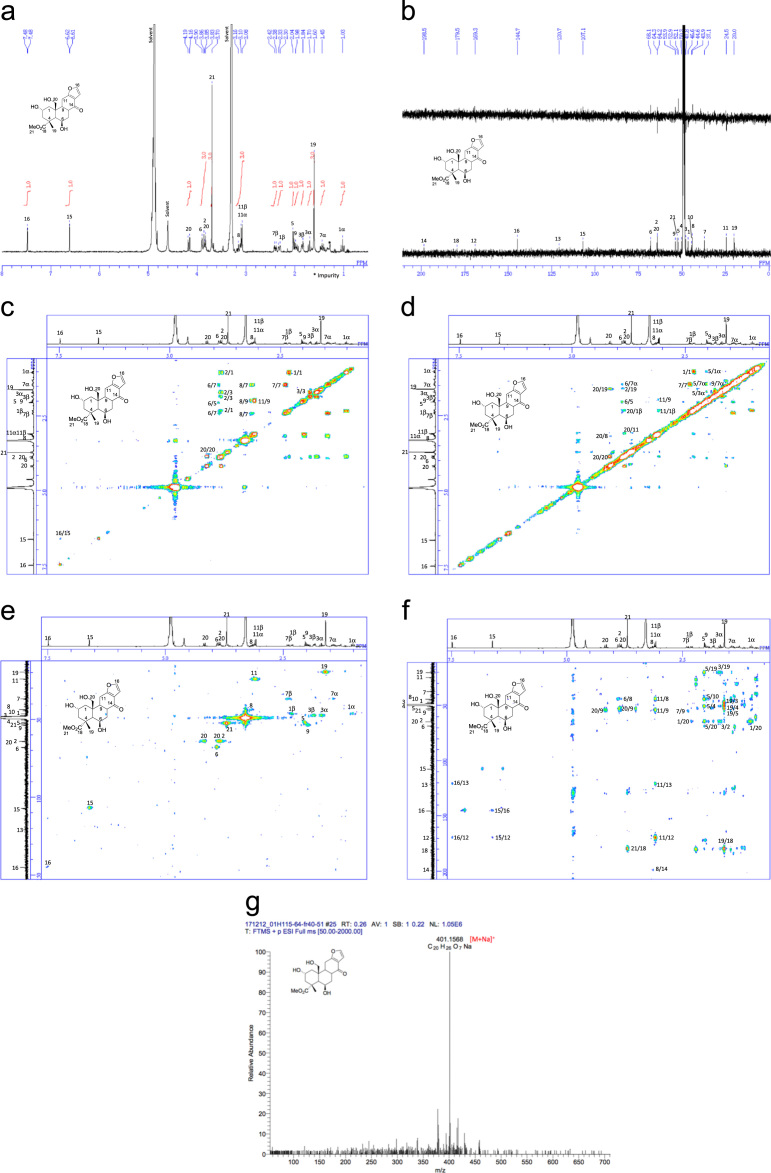
**a.**^1^H NMR (400 MHz, CD_3_OD) of **11. b.**^13^C NMR and DEPT (100 MHz, CD_3_OD) of **11. c.**^1^H-^1^H COSY of **11. d.** NOESY of **11. e.** HSQC of **11. f.** HMBC of **11. g.** (+)HRESIFTMS of **11**.

The data set presented in this article focuses on characterization of the cassane-type furanoditerpenoids described in [1]. The article provides the information on the high-performance liquid chromatography (HPLC) profiles and spectroscopic data of the isolated furanoditerpenoids. The HPLC profiles of the furanoditerpenoids **1**–**4** shown in Fig. 1 isolated from the EtOAc-soluble fraction of the intact seeds of *Caesalpinia decapetala* are given in Fig. 2 [2], [3], [4]. The HPLC profiles of the furanoditerpenoids **5**–**11** shown in Fig. 3 isolated from the EtOAc-soluble fraction of the pupal cases produced by *Sulcobruchus sauteri* are given in Fig. 4. Figs. 5a–g, 6a–g, 7a–g, 8a–g, 9a–g, 10a–g, and 11a–g show 1D NMR, 2D NMR, and HRESIFTMS of the undescribed furanoditerpenoids **5**–**11**. Analyses of the spectra of **5**–**11** are described in the research article [1].

## 2 Experimental design, materials, and methods

NMR spectra were acquired using a JEOL A400 spectrometer (400 MHz for ^1^H, 100 MHz for ^13^C). ^1^H and ^13^C NMR chemical shifts were referenced to residual solvent peaks: *δ*_H_ 3.30 (residual CHD_2_OD) and *δ*_C_ 49.0 for CD_3_OD. HRESIMS were carried out using a Thermo Fisher Scientific LTQ Orbitrap XL mass spectrometer at the Natural Science Center for Basic Research and Development (N-BARD), Hiroshima University. HPLC-photodiode array (PDA) analyses were performed with an Inertsil ODS-3 column (150×4.6 mm i.d., 5 μm) on a JASCO LC-2000 instrument equipped with a JASCO MD-2015 multiwavelength detector. The solvents, (A) CH_3_CN and (B) 1% AcOH, were used as the mobile phase in the following gradient elution: 0–5 min, 60% A; 5–45 min, 60–80% A; 45–55 min 80–100% A with a flow rate of 0.6 ml/min.

## 3 Furanoditerpenoids 5–11

### 3.1 Methyl (2S,4R,4aS,6aS,7S,11aR,11bR)-1,2,3,4,4a,5,6,7,11,11a-decahydro-2,7-dihydroxy-4,7-dimethyl-12-oxo-6a,11b-(epoxymethano)phenanthro[3,2-b]furan-4-carboxylate (caesalsauteolide) (5)

1D NMR, 2D NMR, and HRESIFTMS spectra of the compound **5** are shown in Fig. 5a–g.

### 3.2 (2S,4R,4aS,6aS,7R,11aS,11bS)-1,2,3,4,4a,5,6,6a,7,11,11a,11b-Dodecahydro-2-hydroxy-4-(methoxycarbonyl)-4,7-dimethylphenanthro[3,2-b]furan-11b-carboxylic acid (2-hydroxycaesaljapin) (6)

1D NMR, 2D NMR, and HRESIFTMS spectra of the compound **6** are shown in Fig. 6a–g.

### 3.3 (2S,4R,4aS,6S,6aS,7R,11aS,11bS)-1,2,3,4,4a,5,6,6a,7,11,11a,11b-Dodecahydro-2,6-dihydroxy-4-(methoxycarbonyl)-4,7-dimethylphenanthro[3,2-b]furan-11b-carboxylic acid (2,7-dihydroxycaesaljapin) (7)

1D NMR, 2D NMR, and HRESIFTMS spectra of the compound **7** are shown in Fig. 7a–g.

### 3.4 Methyl (2R,4R,4aR,5R,6aS,7R,11aS,11bS,12S)-2,3,4,4a,5,6,6a,7,11,11a-decahydro-2,12-dihydroxy-4,7-dimethyl-1H-5,11b-(epoxymethano)phenanthro[3,2-b]furan-4-carboxylate (2-hydroxycaesalacetal) (8)

1D NMR, 2D NMR, and HRESIFTMS spectra of the compound **8** are shown in Fig. 8a–g.

### 3.5 Methyl (2R,4R,4aR,5R,6aR,11aS,11bS)-1,2,3,4,4a,5,6,6a,7,11,11a,11b-dodecahydro-2,5-dihydroxy-11b-(hydroxymethyl)-4-methyl-7-methylenephenanthro[3,2-b]furan-4-carboxylate (caesalsauterol) (9)

1D NMR, 2D NMR, and HRESIFTMS spectra of the compound **9** are shown in Fig. 9a–g.

### 3.6 Methyl (2R,4R,4aR,5R,6aR,11aS,11bS)-5-acetoxy-1,2,3,4,4a,5,6,6a,7,11,11a,11b-dodecahydro-2-hydroxy-11b-(hydroxymethyl)-4-methyl-7-methylenephenanthro[3,2-b]furan-4-carboxylate (6-acetylcaesalsauterol) (10)

1D NMR, 2D NMR, and HRESIFTMS spectra of the compound **10** are shown in Fig. 10a–g.

### 3.7 Methyl (2R,4R,4aR,5R,6aR,11aS,11bS)-1,2,3,4,4a,5,6,6a,7,11,11a,11b-dodecahydro-2,5-dihydroxy-11b-(hydroxymethyl)-4-methyl-7-oxophenanthro[3,2-b]furan-4-carboxylate (norcaesalsauterol) (11)

1D NMR, 2D NMR, and HRESIFTMS spectra of the compound **11** are shown in Fig. 11a–g.
