# Supplementary material for: HPLC profiles and spectroscopic data of cassane-type furanoditerpenoids
Source: Data Brief. 2018 Oct 25;21:1076–88. doi: 10.1016/j.dib.2018.10.068 (PMC6226569; doi:10.1016/j.dib.2018.10.068)
Supplement: Supplementary file 1 — Supplementary material. [file mmc1.docx]

**Conflict of Interest**

All authors confirm no conflict of interest.
